# Machine learning in pancreas surgery, what is new? literature review

**DOI:** 10.3389/fsurg.2023.1142585

**Published:** 2023-06-13

**Authors:** Anas Taha, Stephanie Taha-Mehlitz, Niklas Ortlieb, Vincent Ochs, Michael Drew Honaker, Robert Rosenberg, Johan F. Lock, Martin Bolli, Philippe C. Cattin

**Affiliations:** ^1^ Department of Biomedical Engineering, Faculty of Medicine, University of Basel, Allschwil, Switzerland; ^2^ Clarunis, Department of Visceral Surgery, University Center for Gastrointestinal and Liver Diseases, St. Clara Hospital and University Hospital, Basel, Switzerland; ^3^ Goethe University Frankfurt, Faculty of Business and Economics, Frankfurt am Main, Germany; ^4^ Department of Surgery, East Carolina University, Brody School of Medicine, Greenville, NC, United States; ^5^ Cantonal Hospital Basel-Landschaft, Centre for Gastrointestinal and Liver Diseases, Liestal, Switzerland; ^6^ Department of General, Visceral, Transplantation, Vascular and Pediatric Surgery, University Hospital Würzburg, Würzburg, Germany

**Keywords:** machine learning, deep learning, pancreas surgery, scoping review, pancreas

## Abstract

**Background:**

Machine learning (ML) is an inquiry domain that aims to establish methodologies that leverage information to enhance performance of various applications. In the healthcare domain, the ML concept has gained prominence over the years. As a result, the adoption of ML algorithms has become expansive. The aim of this scoping review is to evaluate the application of ML in pancreatic surgery.

**Methods:**

We integrated the preferred reporting items for systematic reviews and meta-analyses for scoping reviews. Articles that contained relevant data specializing in ML in pancreas surgery were included.

**Results:**

A search of the following four databases PubMed, Cochrane, EMBASE, and IEEE and files adopted from Google and Google Scholar was 21. The main features of included studies revolved around the year of publication, the country, and the type of article. Additionally, all the included articles were published within January 2019 to May 2022.

**Conclusion:**

The integration of ML in pancreas surgery has gained much attention in previous years. The outcomes derived from this study indicate an extensive literature gap on the topic despite efforts by various researchers. Hence, future studies exploring how pancreas surgeons can apply different learning algorithms to perform essential practices may ultimately improve patient outcomes.

## Introduction

### Rationale

Machine learning (ML) is an inquiry domain that aims to establish methodologies that leverage information to enhance performance on various applications. ML is an essential branch of artificial intelligence that helps in solving traditionally-complex challenges (1). In the healthcare domain, the ML concept has gained a lot of prominence over the years. The adoption of ML algorithms has become numerous. Qayyum et al. claimed that healthcare professionals could use ML in prognosis, diagnosis, treatment, and clinical workflows (2). Pancreatic surgery represents a field of surgery whose primary purpose is to resect malignant tumors in an effort to improve patient survival. Thus, the analysis of ML's contribution to pancreatic surgery is vital to understanding the algorithm's various benefits, shortcomings, and future expectations. The aim of the study is to provide additional information concerning the utilization of ML in pancreatic surgery, facilitating the development of more algorithms in the field.

Previous researchers in the field have found a positive association between the application of machine learning algorithms and success in pancreatic surgery. For example, Dalal et al. revealed that radiomics demonstrated promising outcomes in the diagnosis and prognosis of pancreatic cystic lesions (3). From a similar perspective, Zhou et al. indicated that ML-based approaches had a higher prediction capacity to the point of outperforming other conventional models used in predicting acute pancreatitis (4). Additional studies have shown that machine learning supported decision derivation in the personalized oversight of pancreatic cancer (5). Furthermore, Palumbo et al. established that ML algorithms was a significant prediction tool for probability of recurrence in pancreatic adenocarcinoma (6). This study offers a scoping review of the relevant publications in the field of pancreatic surgery to aid in developing sound conclusions.

### Objectives

The primary goal of this scoping review is to evaluate the application of ML in pancreatic surgery. The following objectives were adopted to fullfil this aim:

• Evaluating the current application of machine learning in pancreatic surgery
• Analyzing the benefits of integrating machine learning in pancreatic surgery.
• Examining the future of the application of machine learning in pancreatic surgery.

## Methods

### Protocol

This study followed the Preferred Reporting Itens for Systematic Reviews and Meta-Analysis Extension for Scopic Reviews (PRISMA-ScR) guidelines (7). The technique has twenty compulsory features and two optional elements that are utilized in scoping reviews to ensure quality results. PRISMA-ScR was applied as it will allow assessment of the pros and cons of the study to take place. Also, the process will facilitate the duplication of review techniques used in future studies when analyzing the topic. More importantly, the PRISMA-ScR technique will ensure transparency and fairness in the current study (8, 9). According to Sarkis-Onofre et al., the process facilitates the amplification of the methods researchers use in their studies, airing the results and developing research strategies (10). As a result, the PRISMA-ScR principle assists in presenting the techniques used to acquire outcomes.

### Eligibility criteria

The relevancy of the study determined the type of eligibility criteria implemented articles published between 2019 and May 2022 containing relevant data specializing in ML in pancreas surgery and those with original study design undertaken on human participates were included This timeframe was used to guarantee gathering of recent, updated information concerning the application of ML in pancreas surgery. Articles specializing in other forms of surgery were excluded.

### Information sources

Choosing the suitable files for the research topic entailed performing a comprehensive literature scan of different databases and search engines. Databases utilized included PubMed, Cochrane, EMBASE, and IEEE, while search engines included Google and Google Scholar. We explicitly searched for articles printed from 2019 to 2022. The literature search was performed in May 2022. Two team members with experience in the techniques utilized took part in refining the adopted search strategies. An equal chance to voice opinions in a group discussion was undertaken. Subsequently, we evaluated the references of relevant journals to access different properties that were fit for inclusion. We also conducted a search of appropriate journals to add to the study, utilizing Google and Google Scholar search engines. The amalgamation of this approach endorsed a broad literature search, thereby ensuring the gathering of additional supporting information on the study topic.

### Search

The search strategy used in the study entailed keying in specific terms in the relevant search engines and databases. On the PubMed, Cochrane, EMBASE, and IEEE databases, the words used included “machine learning in pancreas surgery,” “benefits of machine learning in pancreas surgery,” “definition of machine learning,” and “disadvantages of machine learning in pancreas surgery.” Filters used on PubMed were “best match” and “2019 to 2022.” On Cochrane, we used the date filter to certify that the articles gathered were published from 2019 to 2022 and the trials filter to guarantee acquired publications were original studies. On EMBASE, the researchers integrated the “study type” and “publication year” filters to gather relevant documents used in the research. We used the “2019–2022” filter on IEEE to gather relevant documents. On Google and Google Scholar search engines, phrases used included “machine learning in pancreas surgery,” “benefits of machine learning in pancreas surgery,” and “definition of machine learning”. We did not incorporate any filters on Google, though, on Google Scholar, the filters incorporated included “2019 to 2022,” “sort by relevance,” and “any type.” A librarian was in charge of the oversight search method as well as enlisting the search strategy. The additional team members counter-checked the process to guarantee validity and reliability by implementing the Peer Review of Electronic Search Strategies (PRESS) checklist. According to Bramer et al., the PRESS agenda directs researchers to check the search strategies included in the study (11). As a result, this method enabled us to show that the foundations attained echoed a precise understanding of the research question.

### Selection of evidence sources

Choosing sources of extracting evidence necessitated focusing on the screening process. All publications were screened to guarantee that they were complete scripts and had the necessary information to qualify for inclusion. We matched the abstracts and complete texts to make sure they fitted. We exluded cases of an included article lacking complete scripts as it would be challenging to establish reliable conclusions. Workload was reduced during screening by first assessing files from all platforms before the process.

### Data charting

We created a data charting form and outlined aspects to be observed when gathering information. The form emphasized elements such as ML in pancreas surgery.

### Data items

Acquisition of information was based on various backgrounds of articles. For example, the papers addressed the medical field and emphasized spreading awareness of ML in pancreas surgery.

### Synthesis of results

For this research, we arranged the journals according to their primary area of concentration. The identified category was ML in pancreas surgery.

### Critical appraisal of results

We used the Measurement Tool to Assess Systematic Reviews (AMSTAR) technique to analyze the operational quality used in the incorporated studies that facilitated settlement of any disagreements among the authors. Moreover, the tool guarantees reliable satisfaction of different systematic reviews and randomized controlled treatment trials (12). Furthermore, there are eleven items in the tool the researcher should use to know the quality of the articles. The technique ensured the implemented studies fulfilled and met the needs of the criterion.

## Results

### Selection of evidence sources

**Figure 1:**
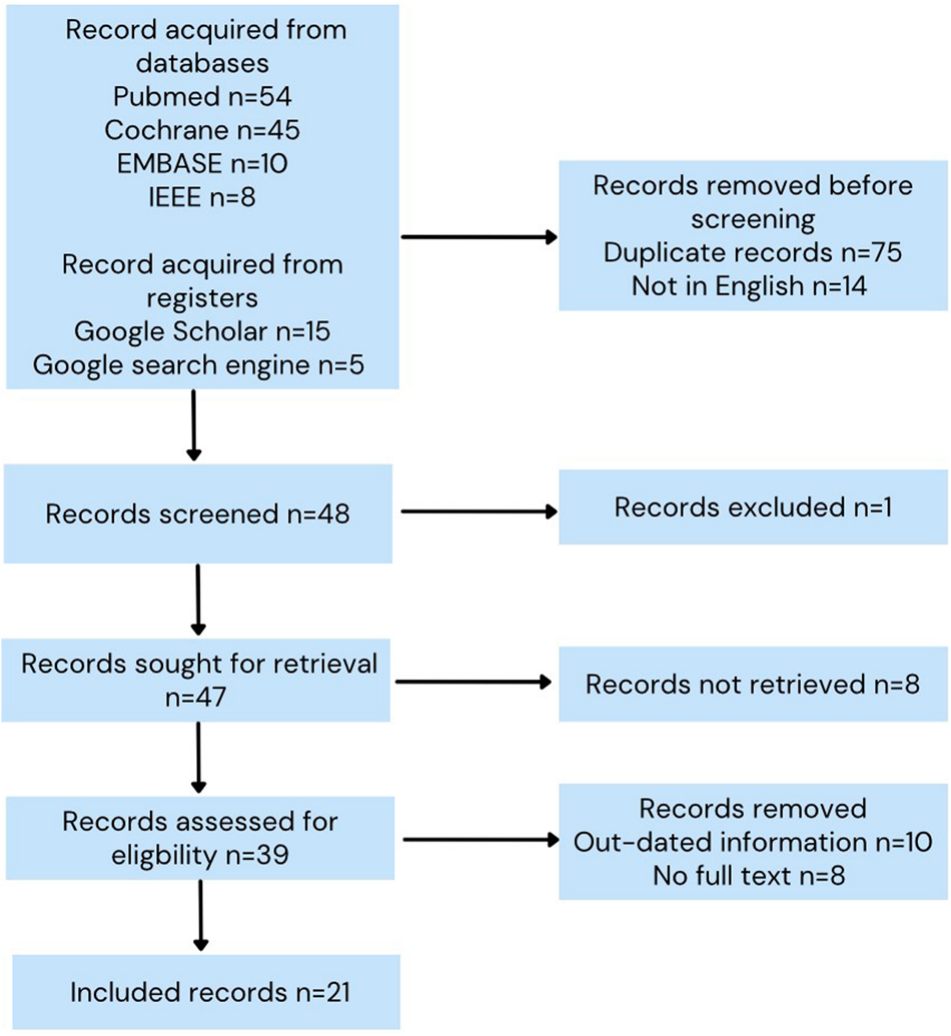
Prisma flowchart.

The search on the four databases adopted (PubMed, Cochrane, EMBASE, and IEEE) yielded 117 results. The number of files acquired from Google and Google Scholar was twenty. Out of the included articles, there were seventy-five duplicate documents. The screening process identified fifty-three articles eligible for inclusion. After seeking retrieval and assessing eligibility, only twenty-one articles were deemed appropriate for inclusion Figure 1. The main themes of the included studies revolved around ML integration in pancreas surgery. Most studies tested machine learning's prediction abilities in the sector (Table 1).

**Table 1:** Main themes of included studies and characteristics.

Author/Years	Country	Primary Theme
Kang et al. (13) 2020	South Korea	ML algorithm was successful in predicting risk of pancreatic malignant intraductal papillary mucinous neoplasm (PDAC)
Springer et al. (14) 2021	USA	CompCyst showcased success in guiding the management of pancreatic cysts
Awe et al. (15) 2022	USA	XGBoost successfully predicted mucinous pancreatic cysts using compted tomography radiomics
Qiu et al. (16) 2019	Multiple countries	ML accurately predicted the histopathological grade of PADC
Kaissis et al. (17) 2019	Germany	XGBoost accurately identified the various subtypes of PDAC
Savareh et al. (18) 2020	Iran	ML demonstrated success in diagnosing pancreas cancer using microRNAs
Kaissis et al. (19) 2020	Germany	ML successfully predicted molecular PDAC subtypes essential for patient treatment
Wang et al. (20) 2021	China	ML can facilitate the early detection of PDAC
Lan et al. (21) 2020	Multiple countries	ML showcased success in predicting surgical intervention timing for necrotizing pancreatitis
Merath et al. (22), 2020	USA	Decision tree models predicted 13 out of 17 complications among pancreatic, liver, and colorectal surgery patients
Kambakamba et al. (23) 2020	Switzerland	ML-based texture analysis assisted in predicting postoperative pancreatic fistula
Cos et al. (24) 2021	USA	ML successfully predicted postoperative morbidity among pancreatectomy patients
Pfitzner et al. (25) 2021	Germany	Logistic Regression was the best performer in predicting perioperative risk
Qu et al. (26) 2020	China	XGBoost demonstrated high performance in predicting acute kidney injury among pancreatitis patients
Yokoyama et al. (27) 2021	Japan	Support vector machine and neural network successfully predicted survival of pancreatic cancer patients after surgery
Iwatate et al. (28) 2020	Japan	Radiogenomic could successfully predict p53 mutations among patients
Toyama et al. (29) 2020	Japan	Radiomics with random forest were successful in enhancing prognosis among pancreatic cancer patients
Baig et al. (30) 2021	Canada	Supervised ML helps with prognosis among patients
Hayashi et al. (31), 2022	Japan	ML-based algorithms were successful in predicting recurrence of pancreatic cancer
Sala-Elarre et al. (32) 2019	Spain	ML algorithms successfully predicted the risk of relapse among pancreatic cancer patients
Li et al. (33) 2021	China	ML algorithms predicted relapse risk

### Study characteristics

The main features of included studies revolved around the year of publication, the country, and the type of article. The majority of articles incorporated into this study were journal articles (*n* = 21). Only one conference proceedings paper was included. Additionally, the publication of all included articles occurred between 2019 to May 2022. Articles selected discussed various ML algorithms, including linear regression, support vector machine, and extreme gradient boosting (XGBoost). Details of included articles are displayed in Table 2.

**Table 2:** Data types, sizes, and evaluation metrics of included articles.

Author	Datatype	Dataset-Size	Test -Size	ML Type	Evaluation Metrics
Kang et al. (13)	Float	*n* = 3708	*n* = 3708	Extreme gradient boosting (XGBoost), deep learning, distributed random forest, generalized linear model, gradient boosting machine (GBM), extremely randomized trees, and stacked ensemble	Performance outcome of 72,5%
Springer et al. (14)	Integer	*n* = 875	*n* = 862	CompCyst	Successful performance
Awe et al. (15)	Float	*n* = 99	*n* = 99	XGBoost	Success in developing a classifier
Qiu et al. (16)	Integer	*n* = 132	*n* = 56	Support vector machine (SVM)	86% accuracy, sensitivity of 78%, and 59% specificity.
Kaissis et al. (17)	Integer	*n* = 102	*n* = 55	XGBoost	Excellent performance
Savareh et al. (18)	Float	*n* = 671	*n* = 671	Particle Swarm Optimization, Artificial Neural Network, and Neighborhood Component Analysis	93% accuracy and sensitivity and 92% specificity
Kaissis et al. (19)	Float	*n* = 207	*n* = 207	Random forest (RF)	84% sensitivity
Wang et al. (20)	Float	*n* = 1033	*n* = 1033	Greedy algorithm and SVM	86% accuracy
Lan et al. (21)	Float	*n* = 223	*n* = 183	Logistic regression (LR), SVM, and RF	Successful prediction of surgical intervention timing
Merath et al. (22)	Float	*n* = 15675	*n* = 15675	Decision tree model (DT)	C-statistic of 74%
Kambakamba et al. (23)	Integer	*n* = 110	*n* = 110	k-nearest neighbors (KNN), C5.0, sequential minimum optimization, multilayer perception, RF	96% sensitivity and 98% specificity
Cos et al. (24)	Float	*n* = 54	*n* = 48	RF, gradient boosted trees, KNN, SVM with linear kernel, and LR	Performance AUROC curve of 78%
Pfitzner et al. (25)	Float	*n* = 521	*n* = 521	LR with L2 regularisation, DT, SVM, GBM, and a combination of feed-forward neural network and rated recurrent unit	AUPRC of 51% for death prediction and 53% for the prediction of significant difficulties
Qu et al. (26)	Float	*n* = 334	*n* = 334	SVM, RF, classification and regression tree and XGBoost	XGBoost demonstrated the highest performance
Yokoyama et al. (27)	Float	*n* = 191	*n* = 191	SVM and neural network	Successfully distinguished patients with pancreatic cancer
Iwatate et al. (28)	Float	*n* = 140	*n* = 107	Mann–Whitney *U* test and XGBoost	Success in predicting p53 mutations
Toyama et al. (29)	Integer	*n* = 161	*n* = 138	Radiomics with RF	Provided vital prognosis data
Baig et al. (30)	Float	*n* = 113	*n* = 93	SVM, RF, and Naïve Bayes	SVM attained a 75% accuracy, 41% sensitivity, and a specificity of 97.5%
Hayashi et al. (31)	Float	*n* = 524	*n* = 524	Convolutional Neural Network and RF	1.000 predictive accuracy
Sala-Elarre et al. (32)	Float	*n* = 40	*n* = 36	LR, Decision Tree, RF, SVM and KNN	Success in predicting relapse risk
Li et al. (33)	Float	*n* = 262	*n* = 183	RF, SVM and KNN	The SVM was the best-performed method

## Discussion

### Summary of evidence

Multiple articles have explored the integration of ML in pancreatic surgery. One of the main domains of ML is prediction, which studies implemented in diagnostics for pancreatic diseases. Kang et al., found that the pancreatic surgery domain applies ML techniques after comparing the performance of ML and logistic regression in predicting the risk for pancreatic malignant intraductal papillary mucinous neoplasm using preoperative clinical data and radiological features (13). In this international multicenter study, patient variables of 3708 subjects were included. The ML method developed consisted of a combination of numerous algorithms such as XGBoost, deep learning, distributed random forest, generalized linear model, gradient boosting machine, randomized trees, and stacked ensemble. The results indicated that both models, logistic regression and ML models, demonstrated similar performance outcomes. The authors asserted that logistic regresion demonstrated higher practicability and interpretability than the ML algorithm (13).

Springer et al. demonstrated the benefits of applying a ML algorithm in pancreas surgery by demonstrating its capability to improve the management of patients with pancreatic cysts (14). The researchers established the CompCyst classifier to suggest various approaches to managing cysts. The study's outcome indicated that the ML algorithm prompted the sparing of surgery among half of the patients who had undergone unnecessary cyst resection. Surgeons can use such machine algorithms to minimize morbidity and the extensive economic expenses of current management practices (14). From a synonymous perspective, Awe et al. examined the integration of ML in pancreatic surgery to predict the occurrence of mucinous pancreatic cysts through the integration of computed tomography (CT) radiomics (15). The specific ML algorithm implemented was XGBoost, which facilitated the generation of mucinous classifiers *via* texture characteristics or radiological and clinical combined models. The results indicated that ML could aid in pancreatic cyst identifiecation by enabling the creation of suitable classifiers (15). These arguments insinuate that many researchers will continue to explore ways to exploit ML techniques to their advantage.

Moreover, further research spearheaded by Qiu et al. revealed that ML was applicable in the pancreatic surgery domain in predicting the histopathological grade of pancreatic ductal adenocarcinoma (PDAC) among patients using preoperative CT scans (16). The tested data size was 56 patients, whereas the adopted ML algorithm was the support vector machine. After a thorough texture analysis, the ML technique achieved an accuracy of 86%, a sensitivity of 78%, and a specificity of 95% (16). Hence, the success of the support vector machine in making predictions about histopathological subtypes among PDACs indicates that the application of ML algorithms will benefit medical practitioners in individualizing treatment options for patients undergoing pancreas surgery. Kaissis et al. also showed the that ML radiomics could facilitate the prediction of molecular subtypes in PDAC (17). The authors aimed to establish a supervised ML approach for predicting the subtypes using diffusion-weighted-imaging-derived radiomic feature characteristics. The ML model used was XGBoost, and it demonstrated success in predicting the various subtypes of PDAC (17). Accordingly, ML integration and consideration into pancreas surgery are likely to increase since knowing the multiple subtypes of PDAC is vital to predict patient survival, response to chemotherapy, and recurrence-free survival.

Regarding diagnostics, Savareh et al. demonstrated that ML could aid in diagnosis of pancreatic cancer *via* circulating microRNA signatures (18). The ML algorithms used in this study were Particle Swarm Optimization, Artificial Neural Network, and Neighborhood Component Analysis. The outcomes illustrated that the developed algorithm showcased 93% accuracy and sensitivity and a 92% specificity (18). This factor implies that the future application of ML in the field is inevitable. In a different study, Kaissis et al. evaluated how pancreas surgeons can apply ML and radiomics to develop a non-invasive approach for facilitating clinical imaging (19). The authors combined features from CT imaging with random forest. The results found that the ML algorithm was influential in predicting the histological phenotyping of PDAC with a sensitivity of 84% (19). Another study by Wang et al. supported this argument highlighting the effectiveness of the greedy algorithm and support vector machine in early detection of PDAC using a combination of metabolomic features. Their approach yielded an 86.74% accuracy and AUC of 0.9351 in the validation cohort of 1,003 patients. Furthermore, in the added prospective collected data of 300 patients, the approach performed with an 85% accuracy and 0.9389 AUC (20). Hence, the approaches were deemed successful in detecting and analyzing the metabolism and systems of PDAC. ML methods may facilitate diagnosis and prediction of malignant pancreatic lesions and even their molecular features. Thus, ML's future application in diagnostics related to pancreas surgery will likely increase in the coming years.

The efficacy of ML models in prediction is also demonstated by the approaches to evaluate the ideal timepoint for surgical intervention. For example, a study conducted by Lan et al. revealed that ML approaches could assist in predicting the timing of surgical intervention among individuals with necrotizing pancreatitis (21). Three ML classifiers were used: logistic regression, support vector machine, and random forest. The results generated *via* the study indicated that ML could be beneficial in predicting the critical features related to surgical timing.

Moreover, the advantages of ML in pancreas surgery are highlighted by studies evaluating postoperative outcomes. Merath et al. assessed how ML algorithms could help predict complications after pancreatic, liver, and colorectal surgeries (22). The researchers used the decision tree models, which accurately forecasted 13 out of 17 complications evaluated. The model demonstrated a significant predictive capability and superiority over previously developed methods. Kambakamba et al. found that ML-based tissue analysis in CT could predict the occurrence of pancreatic fistula preoperatively (23). The pilot study involved 110 patients who had undergone pancreatoduodenectomy from 2008 to 2018. The results reveiled that CT had a sensitivity of 96% and a specificity of 98% in predicting pancreatic fistula after pancreatoduodenectomy (23).

Another study conducted by Cos et al. provides more evidence of ML applications in pancreatic surgery, by predicting postoperative morbidity outcomes among individuals undergoing pancreatectomy through ML and wearable devices (24). The study consisted of 48 patients, using ML methods of random forest, gradient boosted trees, k-nearest neighbors, support vector machine with linear kernel, and logistic regression with L1 penalty. The integrated ML approach utilized patient clinical features and patient activity information to develop the model. The outcomes indicated that the adopted ML algorithm attained the highest performance after scoring an AUROC curve of 0.7875 (24). Further, Pfitzner et al. showed that ML effectively predicts perioperative morbidity and mortality among patients with pancreatic cancer (25). The specific algorithms used were logistic regression, decision tree, support vector machine, gradient boosting machine, and a mixture of feed-forward neural network and rated recurrent unit. The best performing algorithm was logistic regression (25). The results indicated that more interest into ML in pancreas surgery is unavoidable. Furthermore, Qu et al. presented a synonymous argument by stating that pancreas surgeons could integrate ML algorithms to facilitate the prediction of severe kidney injury among individuals with acute pancreatitis (26). The ML model used consisted of a support vector machine, random forest, classification and

regression tree, and XGBoost. Out of all the approaches, the XGBoost demonstrated the best performance in making appropriate predictions after demonstrating an accuracy of 91.93% (26). Thus, it remains evident that machine learning integration into pancreatic surgery could also aid in predicting clinical outcomes.

Yokoyama et al. claimed that ML application in pancreatic surgery could assist in predicting oncological outcomes after resection in patients with pancreatic carcinoma (27). The authors proposed to use tissue samples from individuals with pancreatic neoplasms to assess whether they could act as predictive biomarkers for patients` 5-year overall survival. The integrated ML algorithm was a prognostic classifier formed after integrating support vector technologies, a neural network, and multinomial-based approaches (27). The results supported the approach's success in predicting survival prognosis in their cohort of patients. Different research initiated by Iwatate et al. revealed that ML *via* CT could predict the genetic data for pancreatic tumors in an easy, economical, and non-invasive method *via* cancer imaging analysis (28). The results demonstrated radiogenomic's success in predicting p53 mutations and the consequent prognosis of PDAC patients. Long-term, this could assist in establishing personalized precision treatment by gathering information from CT scans (28). Toyama et al. found that ML algorithms can help in estimating prognosis among pancreatic cancer patients (29). By using radiological features from F-fluorodeoxyglucose-positron emission tomography prior to treatment from 138 patients, results indicated that the ML algorithm adopted provided essential prognostic data by using radiomics only. Similarly, Baig et al. found that ML algorithms could help with outcome prognosis among individuals with cancer of the pancreatic head (30). Their main aim was to analyze whether the ML algorithm developed could successfully predict the survival chances of patients. The authors adopted a supervised ML algorithm which attained a 75% accuracy, 41.9% sensitivity, and 97.5% specificity (30). These studies indicate that ML adoption in pancreatic surgery can lead to multiple positive advantages and the implication has already been broad with oncological outcome prediction in pancreas surgery potentially becoming a domain of ML algorithms.

Additionally, the integration of ML in pancreatic surgery remains evident through the series of publications released on the topic. ML techniques may also be beneficial in forecasting cancer recurrence. For instance, Hayashi et al. aimed to establish and forecast patients undergoing pancreatic surgery initial and late cancer recurrence patterns and matestasis sites using a histology-based ML technique (31). The ML method consisted of a convolutional neural network and random forest combination. The sample involved 524 who had undergone pancreatic cancer surgery between 2001 and 2014. The results indicated that the model had a predictive capacity of 1,000 for nonrecurrence in both the training and test data (31). Moreover, Sala-Elarre et al., claimed that the pancreatic surgery domain could apply ML algorithms in pre-treated patients with pancreatic cancer to help in predicting their risk of developing a recurrence (32). The algorithm developed by the researchers entailed the combination of an intensified induction polychemotherapy with chemoradiation. The results of logistic regression indicated that the algorithm had the potential to predict long-term outcomes and could be a vital tool for predicting the threats of patients relapsing (32). Similarly, a study by Li et al. revealed that ML approaches like random forest, support vector machine and k-neighbor algorithms demonstrated success in predicting the relapse among PDAC patients within a one-year and two-year range using patient-related and histopathological features (33). The results indicated that support vector machine (SVM) had the most accurate approach for predicting relapse among patients who had undergone radical resection (33). Based on these results, it remains evident that pancreatic surgeons are likely to know the various advantages of incorporating ML into the field. Hence, future applications are broad since the algorithms can help guide the establishment of personalized observation systems after surgery.

The increasing integration of ML in pancreas surgery is inevitable due to the efficacy of the models in prediction. Thus, it remains evident that ML integration into pancreatic surgery could help with multiple activities, mainly aiding in diagnostics and prediction of postoperative complications and oncological outcomes.

### Limitations

The study's primary limitation is the paucity of information on the research topic. However, the probable cause for the research gap is the focus on recent articles released from 2019 to 2022. Also, the study exclusively included studies in English, implying that there is a possibility of excluding relevant studies published in other languages.

### Practical and research implications

This research's outcomes are essential as they will improve care of pantients with undergoing pancreatic surgery by suggesting various diagnosis and outcome prediction approaches. Nevertheless, more research into the sector is necessary to ensure recent and accurate information concerning ML incorporation in pancreas surgery.

## Conclusions

The integration of ML in pancreas surgery has gained much attention in previous years. However, the outcomes derived from this study indicate an extensive literature gap on the topic despite efforts by various researchers. Hence, future research should explore how pancreas surgeons can apply different learning algorithms to perform essential practices that ultimately improve patient outcomes. Future studies should also conduct original studies that test ML algorithms' performance using factual data from human participants. This will facilitate increased supporting information that can help delineate the future of ML in pancreas surgery. Further, researchers should explore the various shortcomings associated with ML in pancreas surgery. Mastering the limitations will be essential to developing relevant solutions to the issues. Increased evidence will ensure that surgeons can use ML techniques to make their work easier and guarantee enhanced prediction and diagnosis for pancreatic surgery complications.
